# Supplementary material for: Temperature-Dependent Evolution of Raman Spectra of Methylammonium Lead Halide Perovskites, CH3NH3PbX3 (X = I, Br)
Source: Molecules. 2019 Feb 11;24(3):626. doi: 10.3390/molecules24030626 (PMC6384565; doi:10.3390/molecules24030626)
Supplement: Supplementary file 1 [file molecules-24-00626-s001.pdf]

Supplementary Material

Temperature-Dependent Evolution of Raman Spectra of Methylammonium Lead Halide Perovskites $CH_3NH_3PbX_3$ (X = I, Br)

Kousuke Nakada[1], Yuki Matsumoto[2,†], Yukihiro Shimoi[2], Koji Yamada[3] and Yukio Furukawa[1,*]

[1] Department of Chemistry and Biochemistry, School of Advanced Science and Engineering, Waseda University; Shinjuku, Tokyo 169-8555, Japan; sw-ko@asagi.waseda.jp (K.N.)

[2] Research Center for Computational Design of Advanced Functional Materials (CD-FMat), National Institute of Advanced Industrial Science and Technology (AIST), 1-1-1 Umezono, Tsukuba, Ibaraki 305-8568, Japan; matsumoto.yuki2@mazda.co.jp (Y.M.); y.shimoi@aist.go.jp (Y.S.)

[3] College of Industrial Technology, Nihon University, Izumi-cho 1-2-1, Narashino, Chiba 27-8575, Japan; yamada.kouji@nihon-u.ac.jp

[*] Correspondence: furukawa@waseda.jp; Tel.: +81-3-5286-3244

[†] Present address: Technical Research Center, Mazda Motor Corporation, 3-1 Shinchi, Fuchu-cho, Aki-gun, Hiroshima 730-8670, Japan.

Table S1. Assignments of Raman bands of $CH_3NH_3PbBr_3$

No.	Wavenumber / cm^{-1}				Assignments
	Orthorhombic	Tetragonal I	Tetragonal II	Cubic	
ν_1	328/296	325	326	326	$CH_3NH_3^+$–$PbBr_3^-$ cage vibration
ν_2	916	918	917	916	CH_3 rocking and NH_3^+ rocking
ν_3	971	973	971	969	C–N^+ stretching
ν_4	1259	1254	1253	——	CH_3 rocking and NH_3^+ rocking
ν_5	1421	1424	1423	——	CH_3 symmetric deformation
ν_6	1456	1458	——	——	CH_3 degenerate deformation
ν_7	1461	——	——	——	CH_3 degenerate deformation
ν_8	1472	1482	1479	1478	NH_3^+ symmetric deformation
ν_9	1590	1592	1592	1592	NH_3^+ degenerate deformation
ν_{10}	2820	2823	2824	2829	combination
ν_{11}	2895	2893	2893	——	combination
ν_{12}	2965	2965	2965	2967	CH_3 symmetric stretching
ν_{13}	3032	3034	3036	——	CH_3 degenerate stretching
ν_{14}	3039	——	——	——	CH_3 degenerate stretching
ν_{15}	3105	3105	3107	——	NH_3^+ symmetric stretching
ν_{16}	3144	——	——	——	NH_3^+ degenerate stretching

Table S2. Assignments of Raman bands of $CH_3NH_3PbI_3$

No.	Wavenumber / cm^{-1}			Assignments
	Orthorhombic	Tetragonal	Cubic	
ν_1	~250	258	246	$CH_3NH_3^+$–PbI_3^- cage vibration
ν_2	918	909	908	CH_3 rocking and NH_3^+ rocking
ν_3	969	965	959	C–N^+ stretching
ν_4	——	1249	——	CH_3 rocking and NH_3^+ rocking
ν_5	1422	1421	1423	CH_3 symmetric deformation
ν_6	——	1450	——	CH_3 degenerate deformation
ν_7	——	——	——	CH_3 degenerate deformation
ν_8	1460	1469	1469	NH_3^+ symmetric deformation
ν_9	1587	1583	1582	NH_3^+ degenerate deformation
